# Editorial: Benefiting from microbes: challenges in getting the ‘pros’ and avoiding the ‘cons’

**DOI:** 10.3389/fgstr.2023.1293448

**Published:** 2023-10-24

**Authors:** Santanu Chattopadhyay, Gopal Murugaiyan, Neerja Hajela, Balakrishnan Siddartha Ramakrishna

**Affiliations:** ^1^ Pathogen Biology, Rajiv Gandhi Centre for Biotechnology, Thiruvananthapuram, India; ^2^ Ann Romney Center for Neurologic Diseases, Brigham and Women’s Hospital and Harvard Medical School, Boston, MA, United States; ^3^ Gut Microbiota and Probiotic Science Foundation (India), New Delhi, India; ^4^ Institute of Gastroenterology, SRM Institutes for Medical Science, Chennai, India

**Keywords:** probiotics, nutrition, therapy, antibiotics, dysbiosis, biosensor

Since the first use of the term ‘microbiome’ by Joshua Lederberg, the microbial community in the human body received wide attention from microbiologists and clinicians. Data accumulated on the gut microbiota of healthy and diseased individuals have guided us to revisit the microbial solution of maintaining health through the use of probiotics. Although the credit of recognizing the benefits of gut-friendly microbes goes to Élie Metchnikoff, the consumption of beneficial microbes has existed since ancient times in many cultures. Currently, the consumption of probiotics — well characterized strains of specific microbial species that are ‘generally recognized as safe (GRAS)’ – has become fairly common across cultures. However, our fundamental knowledge of probiotic mechanisms and efficacy is weaker than in many other areas of science and medicine, partly because of the fact that attention on this subject is relatively new. In this editorial we discuss six published papers under the Research Topic “*Benefiting from microbes: challenges in getting the ‘pros’ and avoiding the ‘cons*’”. These papers highlight how probiotics are integral to maintaining high-quality healthy life, prevention of disease, recovery from disease, and also how the status of the gut microbiota can be monitored using inexpensive biosensor devices.

For more than 100 years we have known that the stool of healthy term-born infants is abundant in beneficial lactic acid bacteria and *Bifidobacterium*. These microbes allow newborns to digest the oligosaccharides present in breast milk. Babies born pre-term or those delivered by Cesarean-section typically lack the good microbes in their gut. Brown et al. tested whether enteral supplementation of probiotics would benefit infants suffering from necrotizing enterocolitis (NEC), a serious gastrointestinal emergency affecting very low birth weight (VLBW) infants. They tested the effect of five-strain probiotics (0.5 g of *Bifidobacterium breve* HA- 129, *Lacticaseibacillus rhamnosus* HA-111, *Bifidobacterium bifidum* HA-132, *Bifidobacterium longum* subsp. *infantis* HA-116, and *Bifidobacterium longum* subsp. *longum* HA-135), and found a 67% reduction of NEC (p<0.01) in infants who received the probiotics (N=367) compared to those who did not (N=369).

One of the major determinants of maintaining health is quality nutrition. Eggs, which are abundant sources of high-quality protein, represent cost effective dietary protein options worldwide. *Bacillus amyloliquefaciens* SC06 (BaSC06), a probiotic originally isolated from soil, has the capacity to modulate intestinal flora and immunity in hens, although its effect on the laying hens’ performance and overall health needed further work. Here, Xu et al. report that the strain BaSC06 elevated the abundance of butyrate-producing bacteria in the gut microbiota of hens, resulting in an overall increase in cecal butyrate content and improving the laying performance of the hen as well as egg quality.

Zhou et al. investigated the effect of *Limosilactobacillus reuteri* FN041, a probiotic isolated from breast milk, on atopic dermatitis (AD). The authors induced AD in the ear of BALB/C pups using calcipotriene (MC903) and ovalbumin. They found a reduction in the inflammatory response in the ear of the pups that received FN041 compared to the pups that did not receive it. A proportionate reduction of the spleen regulatory T cells and plasma IgE was also found in the probiotic treated group. This effect was associated with alterations in the abundance of *Limosilactobacillus*, *Faecalibacterium*, *Bifidobacterium*, and *Akkermansia* in the ileum, and changes in the ileal mucosal barrier integrity. Vertical transmission of the FN041 through the dams to the gut of pups seemed to be more effective than direct supplementation to the pups.

The mechanism by which probiotics exert beneficial effects has been of particular interest in the recent past. Probiotics are used not just to maintain health, but also as supplements in the therapy against a disease. In this context, Singh et al. critically evaluated the potential of using probiotics in immunotherapy (e.g. Chimeric Antigen Receptor or CAR therapy) against a number of cancers (e.g. colorectal cancer, liver cancer, breast cancer lung cancer).

Treatment of infectious diseases by antibiotics is known to alter the gut microbiome of the patients. While antibiotics remain a vital tool in medicine, concerns persist regarding their long-term effects on the gut microbiota. In a systematic review, Girma analyzed the impact of eight relatively new antibiotics (Ceftobiprole, ceftaroline, telavancin, dalbavancin, tigecycline, fidaxomicin, MCB3681, and doxycycline) on human gut microbiota. Based on the available data it is apparent that all these antibiotics have significant potential to cause gut dysbiosis.

The impact of the microbial community in the human gut in health is now well appreciated not just by academicians, but also by many informed individuals worldwide. However, people cannot monitor their gut microbiota themselves by point-of-care (PoC) home testing since it requires expensive instrumentation (like next-generation sequencing) and complex bioinformatics analysis. Some of these facilities are not available even in universities and medical colleges with limited resources. Ngashangva and Chattopadhyay have discussed and evaluated the recent trends in biosensor technologies as portable and affordable PoC devices to monitor microbial abundance and metabolic products in the human gut. Recent advancements in technology suggest that, in the near future, it would become possible to monitor gut microbiota health by using portable electronic devices and smartphones.

We have made substantial progress in the recent past and the literature on probiotics is getting richer day by day. However, as was mentioned in the beginning of the editorial, several fundamental aspects regarding probiotic mechanisms of action and efficacy remain poorly addressed. In our opinion, some areas need more attention: a) properly addressing and documenting the risks associated with these ‘GRAS’ microbes; b) improving the shelf-life of the probiotics so that adequate colony forming units of the live microbes can be delivered with each dose; and c) documenting clinical efficacy of probiotics in disease conditions by ensuring that the same product is used across studies in a particular clinical condition, by ensuring that studies are adequately powered, and that hard end-points are used. These will ensure that probiotic use for maintenance of health, and for prevention and amelioration of disease, will be placed on a much firmer scientific footing.

## Author contributions

SC: Conceptualization, Writing – original draft, Writing – review & editing. MG: Writing – review & editing. NH: Writing – review & editing. BR: Writing – review & editing.

